# Transport of a Peptide from Bovine α_s1_-Casein across Models of the Intestinal and Blood–Brain Barriers

**DOI:** 10.3390/nu12103157

**Published:** 2020-10-16

**Authors:** Brian Christensen, Andrea E. Toth, Simone S. E. Nielsen, Carsten Scavenius, Steen V. Petersen, Jan J. Enghild, Jan T. Rasmussen, Morten S. Nielsen, Esben S. Sørensen

**Affiliations:** 1 Department of Molecular Biology and Genetics, Aarhus University, DK-8000 Aarhus, Denmark; bc@mbg.au.dk (B.C.); csss@mbg.au.dk (C.S.); jje@mbg.au.dk (J.J.E.); jatr@mbg.au.dk (J.T.R.); 2 iFood Center, Aarhus University, DK-8000 Aarhus, Denmark; 3 Department of Biomedicine, Faculty of Health, Aarhus University, DK-8000 Aarhus, Denmark; toth@biomed.au.dk (A.E.T.); ssen@biomed.au.dk (S.S.E.N.); svp@biomed.au.dk (S.V.P.); mn@biomed.au.dk (M.S.N.); 4 Interdisciplinary Nanoscience Center, Aarhus University, DK-8000 Aarhus, Denmark

**Keywords:** milk peptides, gastrointestinal digestion, Caco-2 cells, blood–brain barrier, peptide transport

## Abstract

The effect of food components on brain growth and development has attracted increasing attention. Milk has been shown to contain peptides that deliver important signals to the brains of neonates and infants. In order to reach the brain, milk peptides have to resist proteolytic degradation in the gastrointestinal tract, cross the gastrointestinal barrier and later cross the highly selective blood–brain barrier (BBB). To investigate this, we purified and characterized endogenous peptides from bovine milk and investigated their apical to basal transport by using human intestinal Caco-2 cells and primary porcine brain endothelial cell monolayer models. Among 192 characterized milk peptides, only the α_S1_-casein peptide ^185^PIGSENSEKTTMPLW^199^, and especially fragments of this peptide processed during the transport, could cross both the intestinal barrier and the BBB cell monolayer models. This peptide was also shown to resist simulated gastrointestinal digestion. This study demonstrates that a milk derived peptide can cross the major biological barriers in vitro and potentially reach the brain, where it may deliver physiological signals.

## 1. Introduction

Milk comprises a complex and dynamic mixture of macro- and micronutrients that in combination serve as the optimal nourishment for neonates and infants. Beyond being a source of amino acids, many milk proteins and peptides possess important physiological functions. This includes bioactivities, such as regulation of the immune, cardiovascular and nervous systems. Moreover, milk proteins and peptides have been shown to possess antimicrobial and probiotic functions [1,2]. Peptides with such activities are often encrypted within the sequences of milk proteins and released upon proteolysis by endogenous milk proteases or digestive enzymes.

In milk, many smaller peptides with a size of 2–20 amino acid residues have been identified [3,4]. The majority of these peptides originates from the caseins, and in a recent study all but one out of 248 identified peptides in bovine milk were casein derived [4]. Some of these peptides have well-described bioactivities, such as antimicrobial activity, inhibition of the angiotensin converting enzyme and immunomodulation [4].

For milk peptides to exert their potential biological activity in the intestine or elsewhere in the body, they have to resist pepsin activity at a low pH in the stomach. Furthermore, they must at least for a short period of time resist the activity of pancreatic enzymes in the duodenum. Several peptides from caseins have been shown to resist simulated gastrointestinal digestion [5]. Such peptides can potentially exert their physiological effect via interactions with cellular receptors in the small intestine, or they can be transported across the intestinal barrier to the circulation and, from there, they can mediate effects throughout the body. Human colon adenocarcinoma (Caco-2) cell monolayers are widely used as an in vitro model to investigate absorption across the intestinal epithelium [6]. Caco-2 cells spontaneously differentiate in culture to form a confluent monolayer of polarized cells showing structural and functional characteristics similar to mature enterocytes. In particular, differentiated Caco-2 monolayers display cell polarization, microvillus structure, carrier-mediated transport systems and tight junctions, and are therefore considered well suited for studies of intestinal uptake [6,7]. In vitro studies have shown that bioactive milk peptides can cross Caco-2 monolayers, e.g., ACE-inhibitory peptides from β-lactoglobulin and whey hydrolysates [8,9], IgE immunoreactive peptides from simulated infant digestion [10], the opioid peptides β-casomorphin-5 and β-casomorphin-7 [11], two anxiolytic peptides from αs_1_-casein [12] and a 17 residue immunomodulatory peptide from β-casein [13]. In vivo, the caseinomacropeptide (residues 106–169 of κ-casein) has been detected in plasma from newborns after breastfeeding and formula feeding, as well as in adult plasma after milk or yogurt ingestion [14,15]. The milk protein osteopontin resists gastric digestion [16], and osteopontin or fragments hereof have also been detected in the plasma of osteopontin-fed mice and infants [17,18].

In recent years, the nutritional impact on brain development and cognitive function has attracted increasing attention. This includes a search for peptide components that can induce signaling across the blood–brain barrier (BBB) or even cross this barrier and gain access to the brain. The BBB is a highly selective permeable barrier between the blood and the brain parenchyma essential for maintenance of central nervous system homeostasis. The tight BBB can be modelled in vitro using primary porcine brain capillary endothelial cells (PBECs). This well-established PBEC model is highly comparable to the human BBB with regard to high transendothelial electrical resistance (TEER) values, restricted passive permeability and expression of tight junction proteins and transporters specific for brain endothelium [19,20,21]. Based on the TEER values, the model is superior to models based on immortalized human cell lines, and is accepted as being a highly translational model for human studies. Recently, it was shown that a group of opioid peptides, hemorphins derived from hemoglobin, can cross both the intestinal barrier and the BBB in vitro [22]. In vivo studies have shown that casein-derived peptides can be detected in mouse brain tissue after feeding, and furthermore, were reported to prevent cognitive decline in an Alzheimer’s disease model [23]. Likewise, bovine milk osteopontin has been located in the brains of mouse pups after oral gavage [24].

The transport of milk-derived peptides across cell monolayers has primarily been studied using single-peptide systems or in vitro digests of milk proteins [11,13,25]. In this study, in vitro models are used to investigate whether natural endogenous milk peptides can cross both the intestinal epithelium and the BBB. By use of highly sensitive mass spectrometric analyses, we found that out of 192 peptides purified from bovine milk, only a single peptide, ^185^PIGSENSEKTTMPLW^199^ from the C-terminal of α_S1_-casein and its truncated derivatives, ^185^PIGSENSEKTT^195^, ^185^PIGSENSEKTTMP^197^ and ^186^IGSENSEKTTMP^197^, can cross in vitro models of both the intestinal barrier and the BBB. We furthermore show that this peptide is resistant to digestion by gastrointestinal proteases.

## 2. Materials and Methods

### 2.1. Chemicals

All chemicals and reagents were purchased from Merck (Rødovre, Denmark) unless otherwise indicated.

### 2.2. Purification of Milk Peptides

Pooled raw cow’s milk (Danish Holsteins) was obtained from a local farmer directly after morning milking. The milk was kept cooled on ice until purification started. Somatic cell count of the milk was 75,000 cells/mL, indicating good health status. One liter of milk was skimmed by centrifugation at 3000× *g* for 20 min at 4 °C. For removal of caseins and the major whey proteins, the skim milk was heated at 90 °C for 30 min and cooled to room temperature on ice, followed by a pH adjustment to 4.6 with 10% acetic acid. Subsequently, the milk was centrifuged at 3000× *g* for 20 min at 4 °C, and the supernatant containing soluble whey proteins and the endogenous milk peptides were filtered by using an Amicon filter membrane with a 10 kDa cutoff. The flow-through containing peptides and smaller protein fragments was lyophilized. Salt and lactose were removed by reversed phase high pressure liquid chromatography (RP-HPLC). Lyophilized filtrate (150 mg) was dissolved in 0.1% trifluoroacetic acid (TFA) and loaded on a Vydac C_18_ column (The Separations Group, Hesperia, CA, USA) connected to a GE Healthcare LKB system (GE Healthcare, Uppsala, Sweden). The peptides were bound to the column in 0.1% TFA (buffer A) at 40 °C at a flow rate of 0.85 mL/min. Elution of peptides was performed with a gradient of 60% acetonitrile in 0.1% TFA (buffer B) developed over 1 min (0–10 min, 0% buffer B; 10–11 min, 0–80% buffer B; 11–20 min, 80% buffer B). The peptides were detected by measuring the absorbance at 226 nm and subsequently lyophilized. The peptides were quantified by dissolving a sample in phosphate buffer (5 mM NaH_2_PO_4_) and measuring the absorbance at 205 nm.

### 2.3. Synthetic Peptides

The peptide PIGSENSEKTTMPLW from α_S1_-casein was synthesized for use in transport and digestion experiments, and the peptide YDGRGDSVVYGLRSKSKKFRR (N-terminally acetylated and amidated at the C terminal) from human osteopontin was synthesized and used as the digestion control (CASLO, Kgs. Lyngby, Denmark). The sequence, quantity and purity (>90%) of the peptides were verified by the manufacturer. Synthetic peptides (5 mg) were dissolved in 0.1% TFA, divided in smaller aliquots, lyophilized and stored frozen until use.

### 2.4. In Vitro Simulation of Gastrointestinal Digestion

The purified milk peptides and the synthesized PIGSENSEKTTMPLW and YDGRGDSVVYGLRSKSKKFRR peptides were subjected to digestion, essentially as described in [5,26] with few modifications. The peptides were dissolved in 0.15 M of NaCl with a pH of 2.5 at a concentration of 1 mg/mL and incubated with pepsin from porcine gastric mucosa in a 1:100 *w*/*w* enzyme to substrate ratio for 60 min at 37 °C. Before digestion with pancreatic proteases, the sample was lyophilized and washed twice in deionized water. Trypsin and chymotrypsin from a bovine pancreas (Worthington Biochemical Corporation, NJ, USA) (1:100 *w*/*w*) and elastase (Worthington) (1:500 *w*/*w*) were added in 50 mM of ammonium bicarbonate buffer (pH = 7.6) and the mixture was incubated for 60 min at 37 °C. In digests of the synthesized peptides, aliquots were sampled and analyzed after 5 min, 30 min, 60 min and 270 min incubation with the intestinal enzymes. Bovine milk osteopontin was purified as previously described [27] and incubated with the digestive proteases to validate their activity. The digested milk peptides were separated by RP-HPLC on an Aeris C4 widepore column (Phenomenex, Værløse, Denmark) connected to a Shimadzu (Kyoto, Japan) HPLC system. The peptides were separated in 0.1% TFA (buffer A) and eluted with a gradient of 60% acetonitrile in 0.1% TFA (buffer B) at 40 °C. The gradient was developed over 59 min (0–5 min: 0% buffer B; 5–49 min: 0–98% buffer B; 49–59 min: 98% buffer B) at a flow rate of 0.85 mL/min. The peptides were detected by measuring the absorbance at 226 nm and analyzed by matrix-assisted laser desorption ionization time-of-flight mass spectrometry (MALDI-MS).

### 2.5. Cell Cultures

The human colorectal adenocarcinoma cell line, Caco-2, was obtained from DSMZ (Braunschweig, Germany). The cells were grown to 50–60% confluence in Dulbecco’s Modified Eagle Medium (DMEM) Glutamax cell culture medium, supplemented with 10% heat-inactivated fetal bovine serum, 100 units/mL of penicillin and 100 μg/mL of streptomycin (all from Invitrogen, Carlsbad, CA, USA) in a humidified 5% CO_2_/95% air atmosphere at 37 °C. The cells used in the experiments were between passages 30 and 40.

Capillaries were isolated from the brain cortices of domestic pigs 5–6 months old as previously described [20]. PBECs were cultured by plating the capillaries in T75 flasks coated with collagen IV (500 µg/mL) and fibronectin (100 µg/mL) in DMEM-F12 medium, supplemented with 10% plasma-derived serum (First Link, Wolverhampton, UK), heparin (15 U), 100 units/mL of penicillin and 100 µg/mL of streptomycin (PBEC growth medium), and cultured in an incubator with humidified 5% CO_2_/95% air at 37 °C. Puromycin (4 µg/mL) was added to the growth medium for the first three days to obtain a pure culture of PBECs.

Astrocyte conditioned medium was collected every third day from primary rat astrocytes, sterile filtered and frozen until use. Isolation of primary rat astrocytes and preparation of astrocyte conditioned media were carried out as described in [20].

### 2.6. Transfer Studies

The Caco-2 cells used for transport studies were seeded in polyethylene terephthalate culture inserts, with a pore size of 0.4 μm and a growth area of 0.33 cm^2^ at a density of approximately 25,000 cells/insert. The Caco-2 cells were grown for three weeks on the porous membrane before they were used for transepithelial transport. The integrity of the monolayers was confirmed by measuring the TEER using a Millicell ERS-2 volt ohmmeter. Only Caco-2 monolayers with TEER values above 400 Ω cm^2^ were used for the transport analysis.

PBECs were grown until 70–80% confluency as described above and then passaged onto polyester culture inserts with a pore size of 0.4 μm and a growth area of 1.12 cm^2^ (Costar, Corning, NY, USA) at a density of 1.1 × 10^5^ cells/cm^2^ in the PBEC growth medium. In the basolateral chamber, the medium consisted of 1 mL of PBEC growth medium and 0.5 mL of astrocyte conditioned medium. The medium was supplemented with differentiation factors: 550 nM of hydrocortisone, 250 μM of 8-(4-chlorophenylthio)adenosine-3′,5′-cyclic monophosphate (cAMP) and 17.5 μM of RO-201724 two days after seeding. The next day, the integrity of the PBEC monolayer was confirmed by measuring the TEER. Only PBEC monolayers with TEER values higher than 800 Ω cm^2^ were used for the transport analysis.

Following the TEER value measurement, the Caco-2 and PBEC monolayers were gently washed twice with phosphate-buffered saline, and the transport medium (Hanks’ Balanced Salt solution supplemented with 10 mM of HEPES) was added to the apical and basolateral compartments. The inserts were incubated for 15 min or 2.5 h with the purified milk peptides (0.1 mg/mL) or the synthetic PIGSENSEKTTMPLW peptide (2 µg/mL), after which medium from the apical and basolateral compartments were collected for liquid chromatography tandem mass spectrometry (LC-MS/MS) analysis. Inserts incubated with a medium without peptides served as the control in the transport experiments. Membrane integrity was validated by addition of D-[1-^14^C]-Mannitol (3.5 µM, 56.8 mCi/mmol) (Perkin Elmer, Waltham, MA, USA) to the upper chamber of the culture inserts with or without milk peptides for 2.5 h. The passive permeability experiments were performed on four or five individual culture inserts. Basolateral samples were mixed with Ultima Gold liquid scintillation fluid (Perkin Elmer) and counted in a Tri-Carb 2810 TR scintillation counter (Perkin Elmer). The apparent permeability coefficient (Papp, cm/s) was determined as Papp = (dQ/dt)/(AC_0_) where dQ/dt is the steady state flux (cpm/s), A is the surface area of the cell culture insert membrane and C_0_ is the initial concentration in the apical compartment (cpm/mL).

### 2.7. Western Blotting, Immunocytochemistry and Confocal Microscopy

Caco-2 cells grown for three weeks in culture inserts were washed twice with ice-cold PBS and incubated with radioimmunoprecipitation assay buffer (RIPA buffer) for 10 min on ice. The cell lysate was centrifuged for 5 min at 9000× *g*, and the protein content was quantified using a Micro BCA Protein Kit (Thermo Fisher Scientific, Waltham, MA, USA). Cell lysate (50 µg) was separated on 10% NuPAGE Novex bis-Tris precast gels (Invitrogen) according to the manufacturer’s instructions, followed by electroblotting onto a Hybond-P PVDF membrane (GE Healthcare) for immunodetection. The membrane was blocked in 2% Tween in Tris-buffered saline before the addition of a rabbit anti-claudin-1 (Cell Signaling Technologies, Danvers, MA, USA), rabbit anti-occludin or mouse anti-p-120 catenin (BD Bioscience, San Diego, CA, USA) monoclonal antibody (all diluted 1:1000). The proteins were detected with horseradish peroxidase conjugated secondary immunoglobulins with enhanced chemiluminescence on an ImageQuant LAS 4000 instrument (GE Healthcare).

Filter membranes with PBECs grown as described above were fixed with 4% paraformaldehyde in PBS for 10 min at room temperature. The following steps were carried out at room temperature. Subsequently, the cells were permeabilized using 0.1% Triton X-100 in PBS for 10 min and blocked using 2% BSA in PBS with a pH of 7.4 for 30 min. The PBECs were stained with monoclonal mouse anti-claudin-5, polyclonal rabbit anti-ZO-1, polyclonal rabbit anti-occludin (all diluted 1:100 and from Thermo Fisher Scientific) or monoclonal mouse anti-p120-Catenin (1:200) (BD Transduction Laboratory). For detection, the cells were stained with donkey anti-mouse or donkey anti-rabbit Alexa Fluor 568 conjugated secondary antibodies (1:500) (Thermo Fisher Scientific). For nuclei staining, the cells were incubated with Hoechst stain solution (0.125 μg/mL) in distilled water for 10 min. In between the steps, the cells were washed three times in PBS. Finally, membranes were mounted on glass slides using a fluorescent mounting medium (Dako, Glostrup, Denmark).

Cells were observed and imaged using a spinning disk confocal imaging system consisting of an Olympus IX-83 fluorescent microscope with a CSU-X1 confocal spinning disk unit (Yokogawa Electric Corporation, Musashino, Japan), an Andor iXon-Ultra 897 EMCCD camera (Andor, Belfast, UK) and Olympus CellSens software. Z-stack images were obtained using 100X magnification silicone immersion objective (UPlanApo 100xS, 1.35 NA) and processed using Fiji Software. The images are presented as maximum intensity Z-projections, and for each channel, independent brightness and contrast adjustments are applied.

### 2.8. Mass Spectrometry

MALDI-MS was performed using a Bruker Autoflex III instrument (Bruker Daltonics, Bremen, Germany). The analysis was performed using a saturated solution of α-cyano-4-hydroxycinnamic acid. The instrument was operated in reflected positive ionization mode and calibrated in the mass range of 1000 to 3200 Da using a peptide calibration standard (Bruker Daltonics, Bremen, Germany). The theoretical peptide masses were calculated using the General Protein/Mass Analysis for Windows software (Lighthouse Data, Odense, Denmark).

LC-MS/MS analysis was performed on a TripleTOF 6600 mass spectrometer equipped with an Exigent nanoLC 415 system (AB Sciex, Les Ulis, France). All samples were micro-purified using 3 M Empore C18 discs packed in pipette tips [28]. The lyophilized samples were dissolved in 0.1% formic acid, injected on a trap column (2 cm × 100 µm inner diameter) and separated on an analytical column packed in-house in a pulled emitter (15 cm × 75 µm inner diameter). All columns were packed with ReproSil-Pur C18-AQ 3 µm resin (courtesy of Dr. Marisch, Ammerbuch-Entringen, Germany). Peptides were eluted directly into the mass spectrometer with a linear gradient from 5 to 35% solvent B (0.1% formic acid in acetonitrile) for 20 min with a flowrate of 250 nL/min, followed after 10 min with 100% solvent B. Data files were converted to Mascot Generic Format using the Raw Converter (version 1.1.0.18) (http://fields.scripps.edu/rawconv/) or the AB Sciex MS Data Converter beta 1.1 (AB Sciex, Les Ulis, France). The generated peak lists were searched against the Bos taurus proteome (UniProt database, https://www.uniprot.org)) using an in-house Mascot search engine (Matrix Science, London, UK). The searches were made without specifying a protease and with oxidation (Met) and phosphorylation (ST) as variable modifications. Peptide tolerance was set to 10 ppm and MS/MS tolerance was set to 0.2 Da.

### 2.9. Statistics

For each peptide identified by LC-MS/MS, an Expect value was estimated by the search engine, which indicates the likelihood that the observed match between the MS/MS spectra and the peptide sequence is found by chance. Identifications were considered statistically significant at Expect values ≤ 0.05. Only peptides identified in two out of three experiments (Caco-2) or three out of five experiments (PBEC) were considered as transported and included (Table 1 and Table 2). Papp values are presented as mean ± standard deviation of four or five experiments.

### 2.10. Ethical Considerations

All experiments involving animal tissue were performed according to Danish ethical regulations. No animals were sacrificed in the project. The porcine brains used are byproducts from the meat industry and the milk was obtained from a Danish dairy milk farm.

## 3. Results

### 3.1. Purification of Milk Peptides

**Figure 1:**
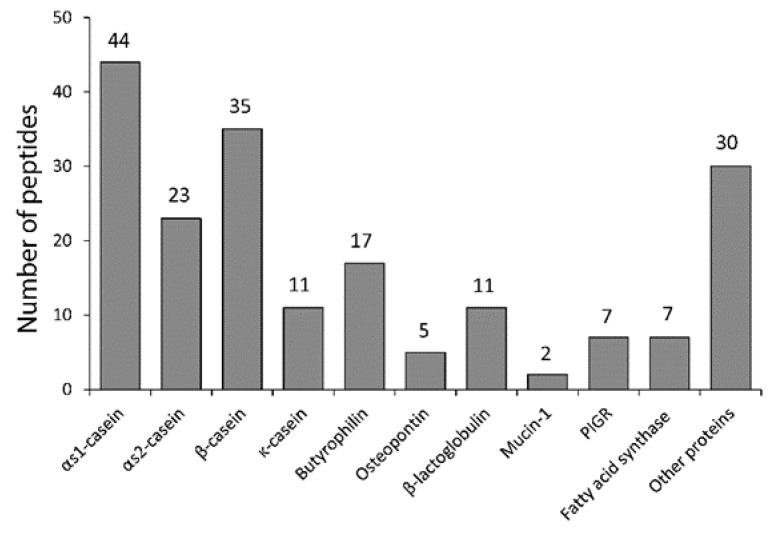
Number of peptides identified from different milk proteins. PIGR, polymeric immunoglobulin receptor.

**Figure 2:**
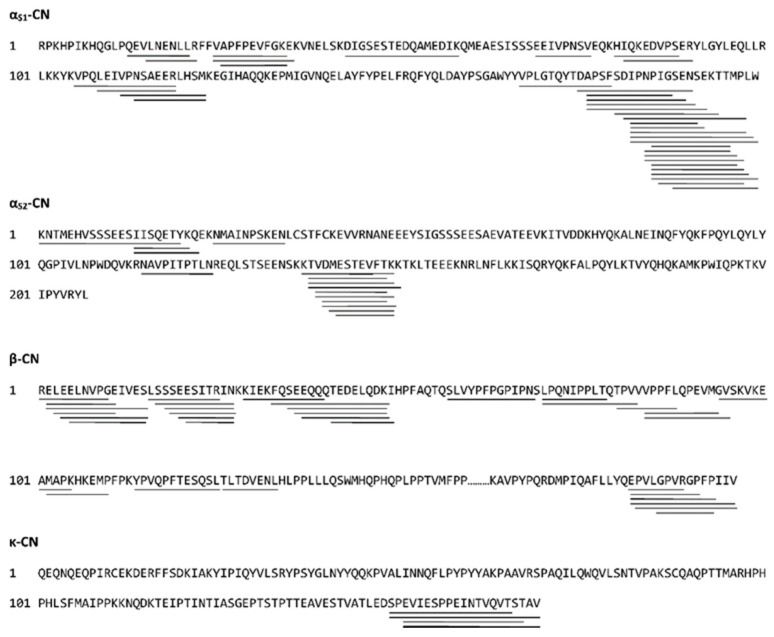
Sequences of the bovine caseins are shown. Lines under the sequences indicate peptides identified by liquid chromatography with tandem mass spectrometry (LC-MSMS) analysis; casein (CN).

Endogenous bovine milk peptides were characterized by LC-MS/MS, resulting in identification of 192 peptides (Table S1). The majority of the peptides originates from the caseins, α_S1_-casein (44), α_S2_-casein (23), β-casein (35) and κ-casein (11) (Figure 1). However, peptides from whey proteins, like osteopontin and beta-lactoglobulin, and proteins from the fat globule fraction, including polymeric immunoglobulin receptor, butyrophilin and fatty acid synthase, were also identified. The identified peptides from the caseins are shown in Figure 2.

### 3.2. Transfer of Milk Peptides Across Caco-2 and PBEC Monolayers

In order to investigate whether milk peptides could cross an in vitro model of the intestinal barrier, they were added to the apical side of differentiated Caco-2 monolayers (representing the intestinal luminal side). After 2.5 h the transcellular transport was monitored by highly sensitive LC-MS/MS analyses of the basolateral media (representing the blood side). Out of a mix of 192 endogenous milk peptides applied to the apical chamber of the Caco-2 cell monolayer, only the α_S1_-casein peptides ^185^PIGSENSEKTTMPLW^199^ and ^185^PIGSENSEKTTMP^197^, the α_S2_-casein peptide ^1^KNTMEHVSSSEESIISQETY^20^ and ^151^EVIESPPEINTVQVTSTAV^169^ from κ-casein were identified in the basolateral chamber (Table 1). The apparent permeability coefficient (Papp) of the Caco-2 monolayer was measured to 9.45 ± 2 × 10^−7^ cm s^−1^, which is indicative of an intact Caco-2 cell monolayer [29]. To further validate the integrity of the model, the expression of tight junction-related and adherens junction proteins was evaluated by western blotting, which showed that Caco-2 cells cultured for 21 days expressed claudin 1, occludin and p120-catenin.

**Figure 3:**
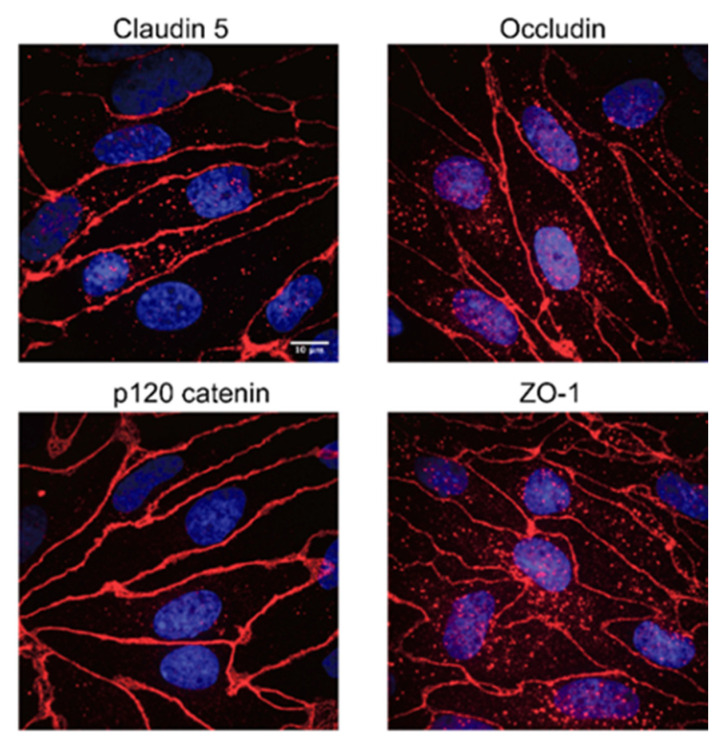
Immunocytochemical analysis of porcine brain endothelial cells (PBECs). Immunofluorescence microscopy analysis of tight junction components Claudin 5, Occludin, ZO-1 and adherens junction protein p120 catenin of PBECs grown on permeable membrane filter inserts with a growth area of 1.12 cm^2^ in the presence of rat astrocyte conditioned medium in the basolateral compartment. Scale bar for all pictures: 10 µm.

In order to investigate whether milk peptides could cross the BBB, an in vitro model using PBECs in a Transwell system was used. The mix of the 192 endogenous milk peptides were applied to the apical compartment (blood side) and after 2.5 h samples from the basolateral media (brain side) were subjected to LC-MS/MS analysis. Peptides from three different proteins were identified in the basal compartment (Table 2). Five independent BBB transport studies were performed, and the α_S1_-casein peptide ^185^PIGSENSEKTTMPLW^199^ or its derived truncated peptides ^185^PIGSENSEKTT^195^, ^185^PIGSENSEKTTMP^197^ and ^186^IGSENSEKTTMP^197^ were identified in all experiments (MS/MS spectra shown in Figure S1. Peptides from β-casein and a polymeric immunoglobulin receptor were identified in three and four experiments, respectively. The Papp for the BBB used in the experiments was 1.09 ± 0.14 × 10^−6^ cm s^−1^, which is level with the lowest obtained Papp value of 0.87 × 10^−6^ cm s^−1^ of other in vitro models of the BBB showing high TEER values up to approximately 2000 Ω cm^2^ [30]. Immunofluorescence analyses of PBECs grown in culture inserts with astrocyte conditioned medium in the basolateral compartment showed localization of the tight junction proteins claudin 5, occludin and ZO-1 along the cell–cell junctions. Likewise, a well-defined distribution along cell–cell junctions of the adherens junction protein p120-catenin was observed (Figure 3).

The transport was also analyzed 15 min after application of milk peptides to the apical compartments of the Caco-2 and the BBB model. These analyses showed no milk peptides in the basolateral compartment.

To investigate whether the α_S1_-casein peptide ^185^PIGSENSEKTTMPLW^199^ was cleaved during transport over the in vitro barriers, the peptide was synthesized and applied to the Caco-2 and PBEC monolayer models. After application of the peptide to the apical chambers, ^185^PIGSENSEKTT^195^ and ^185^PIGSENSEKTTMP^197^ were identified in the basolateral chamber of both monolayers (Table 3).

### 3.3. Digestion of the PIGSENSEKTTMPLW Peptide

**Figure 4:**
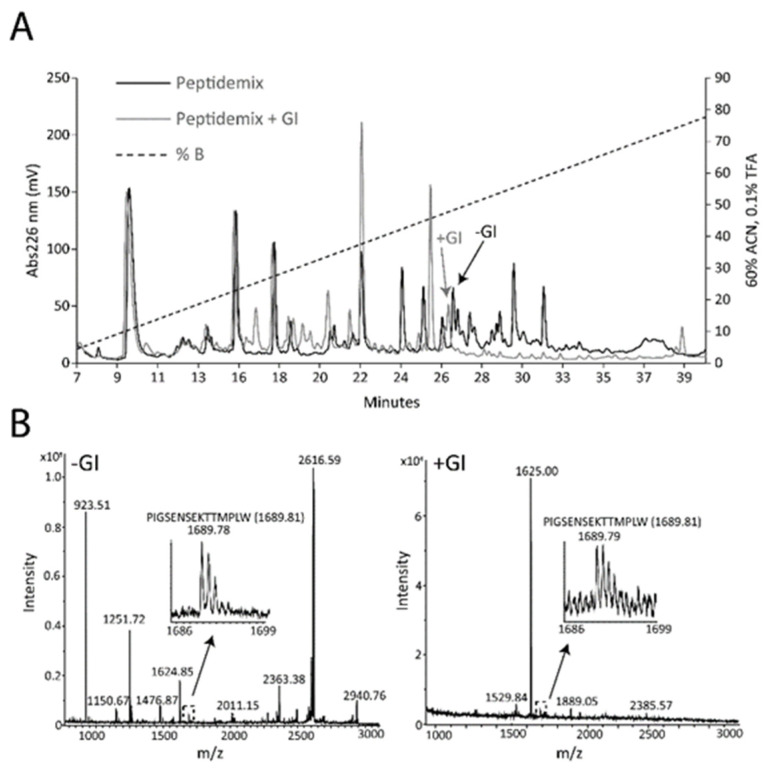
In vitro gastrointestinal digestion (GI) of milk peptides. Milk peptides were incubated with pepsin and then trypsin, chymotrypsin and elastase. (**A**) The peptides were separated by RP-HPLC, eluted with a gradient of 60% acetonitrile (ACN) in 0.1% trifluoroacetic acid (TFA) (dashed line) and detected by measuring the absorbance at 226 nm (solid line). (**B**) All fractions were analyzed by MALDI-MS and representative data from two fractions before and after GI digestion are shown. Both fractions contained the ^185^PIGSENSEKTTMPLW^199^ peptide.

The peptide ^185^PIGSENSEKTTMPLW^199^ was observed to cross both an in vitro intestinal barrier and the BBB model. To test whether the peptide resisted gastrointestinal digestion, the milk peptides were incubated with pepsin and subsequently a mixture of chymotrypsin, trypsin and elastase, and the resulting peptides were separated by RP-HPLC and analyzed by MALDI-MS (Figure 4).

**Figure 5:**
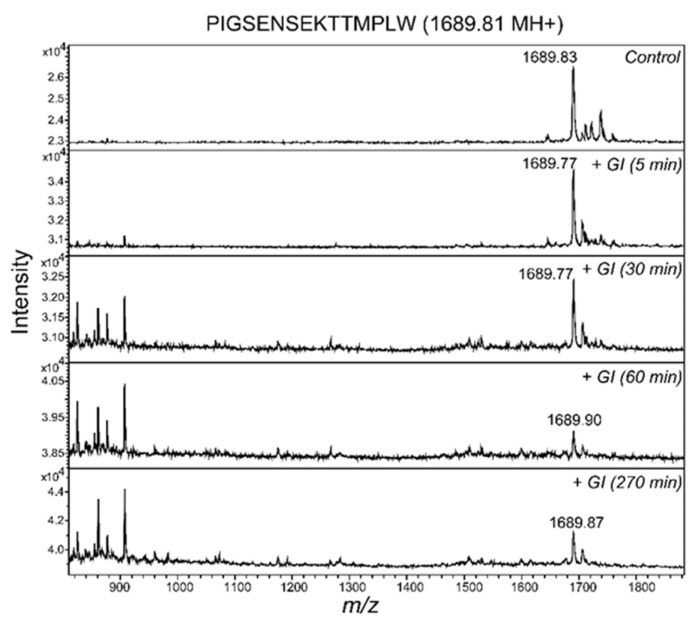
In vitro gastrointestinal digestion (GI) of the PIGSENSEKTTMPLW peptide. The synthetic PIGSENSEKTTMPLW peptide was incubated with pepsin and subsequently with trypsin, chymotrypsin and elastase (for 5 min, 30 min, 60 min and 270 min) to simulate GI digestion. The digests were analyzed by matrix-assisted laser desorption ionization time-of-flight mass spectrometry (MALDI-MS).

In both samples, peptide ions with masses corresponding to ^185^PIGSENSEKTTMPLW^199^ were observed. In addition, the synthetic peptide was digested and analyzed by MS. An ion corresponding to the ^185^PIGSENSEKTTMPLW^199^ peptide was observed even after 270 min of digestion (Figure 5). The activity of the proteases was verified by control digests of another synthetic peptide, YDGRGDSVVYGLRSKSKKFRR, and by digestion of bovine milk osteopontin. The YDGRGDSVVYGLRSKSKKFRR peptide was cleaved after 5 min incubation with the proteases, and likewise, osteopontin was cleaved by all the proteases (Figure S2).

## 4. Discussion

The majority of the identified endogenous milk peptides were derived from the caseins, which is in line with another comprehensive analysis of the native bovine milk peptidome, where 248 peptides were identified and almost exclusively originated from the caseins [4]. The peptides are not evenly distributed throughout the amino acid sequences of the caseins, as they mostly originate from regions that may be more prone to proteolysis by endogenous milk proteases (Figure 2). The peptides derived from α_S1_-casein are mostly from the C-terminal part of the protein (residues 175–199), and the majority of α_S2_-casein peptides are from the sequence region between Lys^137^ and Lys^149^ (Figure 2). Several of the identified peptides contained oxidized methionine residues with an excess mass of 16 Da. These oxidations are most likely artefacts of sample handling and MS analysis [31].

Some of the milk peptides resisted digestion, shown by similar RP-HPLC elution before and after simulated gastrointestinal digestion (Figure 4A). The peptide ^185^PIGSENSEKTTMPLW^199^ from α_S1_-casein was identified after incubation with the gastric protease pepsin and after incubation with the pancreatic enzymes trypsin, chymotrypsin and elastase (Figure 4 and Figure 5), indicating that the peptide can be present in the intestine.

The peptides from α_S1_-casein identified in the basolateral compartment of the BBB model were overlapping peptides consisting of 11–15 amino acid residues with a common core sequence, IGSENSEKTT (Table 2). To investigate whether the different peptides could be generated by proteolytic activity during the transport across the Caco-2 and PBEC monolayers, the PIGSENSEKTTMPLW peptide was synthesized and applied to the apical compartment of the models. After 2.5 h, the basal medium was analyzed by LC-MS/MS and only the two peptides PIGSENSEKTTMP and PIGSENSEKTT were identified (Table 3). This shows that the peptide is cleaved by carboxypeptidases during the transport over the in vitro models of the intestinal barrier and the BBB. The N-terminal proline residue most likely protects the peptides from proteolysis by brush border aminopeptidases. Peptides are highly susceptible to degradation prior to their transport through the intestinal epithelium, and the action of brush border peptidases is important in determining the bioavailability of dietary peptides [32]. Interestingly, the intact PIGSENSEKTTMPLW peptide was observed in the basolateral medium when the mix of milk peptides was applied to the Caco-2 and PBEC monolayers (Table 1 and Table 2). It is plausible that the increased amount of peptide substrates for the indigenous proteases may protect the PIGSENSEKTTMPLW peptide from digestion by substrate competition when the peptide is applied in the mix of milk peptides.

In addition to the peptides from α_S1_-casein, the peptides EPVLGPVRGPFPIIV and PVLGPVRGPFPIIV from β-casein and AAPAGAAIQS and AAPAGAAIQSR originating from the polymeric immunoglobulin receptor were also identified in the basal compartment of the BBB model (Table 2). These peptides were not transported across the Caco-2 monolayers (Table 1), and in five independent BBB transport studies, the peptides from β-casein and the polymeric immunoglobulin receptor were only identified in the basolateral compartment in three and four experiments, respectively, whereas the α_S1_-casein peptide PIGSENSEKTTMPLW or its derived truncated peptides were observed in all experiments. The observation that only very few of the 192 applied peptides are transported across the BBB model emphasizes the specificity of the transport of these specific peptides. In other studies, the β-casein peptide EPVLGPVRGPFPIIV and other variants of this peptide were shown to cross over in vitro intestinal barrier models [13,25]. In these studies, either a much higher amount of a synthetic peptide, YQEPVLGPVRGPFPIIV from β-casein, was applied in the apical compartment [13] compared to our study, or a total of approximately 120 milk peptides were observed to cross the Caco-2 monolayer [25] compared to the few peptides identified in the present study (Table 1).

The peptides present in milk are the results of proteolytic activity of endogenous endo- and exopeptidases. It is not clear which proteases in bovine milk are responsible for the generation of the PIGSENSEKTTMPLW peptide. The proteolytic specificity of the major milk proteases plasmin, cathepsin B, D and G indicate that they are not solely responsible for the release of the peptide from α_S1_-casein [4,33]. However, fermentation of caseins by *Bifidobacterium longum* [34], *Lactobacillus helveticus* [35] and lactic acid starters followed by in vitro gastrointestinal digestion [36] has been shown to release the peptide PIGSENSEKTTMPLW and several of its derivatives observed to cross the intestinal barrier and BBB in this study. This emphasizes the potential relevance of this peptide being transported over physiological barriers, as bovine milk and dairy products constitute a significant part of the diet in many populations.

The PIGSENSEKTTMPLW and IGSENSEKTTMP peptides have previously been identified as potential anti-hypertensive peptides that can inhibit the activity of angiotensin-converting enzyme (ACE) [34,35,36,37]. The ACE-inhibitory ability is presumably mediated through the derived fragments TTMPLW and KTTMP [36,37]. Thus, the transport of this peptide across physiological barriers could influence the regulation of arterial blood pressure. Furthermore, ACE is present in the brain and plays a key role in high salt induced sympatho-excitation, hypertension, neuroprotection, cognition and cerebral vasodilation [38], suggesting that the milk-derived peptides from α_S1_-casein that cross the BBB could potentially take part in ACE regulation in the brain.

A tryptic hydrolysate of bovine milk α_S1_-casein can alleviate sleep disturbances in both animals and humans, most likely via the GABA_A_ receptor [39,40]. The PIGSENSEKTTMPLW peptide contains a C-terminal tryptophan residue, which can be released by proteolysis when the peptide is transported over the BBB (Table 3). Consequently, free tryptophan is released in close proximity to the brain. As tryptophan is the precursor of serotonin, an important neurotransmitter involved in mood and satiety regulation, the release of tryptophan could be speculated to impact physiological and psychological function.

## 5. Conclusions

Out of 192 endogenous milk peptides, only a single peptide, ^185^PIGSENSEKTTMPLW^199^, from the C-terminal of αS1-casein was found to be transported over models of both the intestinal barrier and the blood–brain barrier. The peptide was resistant to digestion by gastrointestinal proteases. The study shows that specific food peptides are capable of crossing physiological membranes in vitro and thus potentially gain access to the blood circulation and even organs like the brain. However, whether this takes place in vivo remains to be shown.

## Figures and Tables

**Table 1:** Milk peptides transported over Caco-2 monolayers identified by Liquid Chromatography with tandem mass spectrometry (LC-MS/MS).

Protein	Peptide	Calculated	Measured	Expect
α_S1_-casein	PIGSENSEKTTMPLW (Mox)	1704.7971	1704.7971	6.00 × 10^7^
	PIGSENSEKTTMP (Mox)	1405.6395	1405.6429	1.60 × 10^4^
κ-casein	EVIESPPEINTVQVTSTAV	2012.0314	2012.0378	1.90 × 10^4^
α_S2_-casein	KNTMEHVSSSEESIISQETY	2314.0271	2314.0082	8.60 × 10^4^
	KNTMEHVSSSEESIISQETY	2298.0321	2298.0254	2.30 × 10^4^

The calculated and measured masses are listed. The shown Expect values are from a representative experiment. Expect values below 0.05 are considered significant. Oxidation of methionine (Mox) is indicated. Only peptides identified in two out of three experiments are considered as transported and included in the table.

**Table 2:** Milk peptides transported over porcine brain endothelial cell (PBEC) monolayers (Blood Brain Barrier model) identified by LC-MS/MS.

Protein	Peptide	Calculated	Measured	Expect
α_S1_-casein	PIGSENSEKTTMPLW (Mox)	1704.8029	1704.7983	9.50 × 10^8^
	PIGSENSEKTT	1161.5513	1161.5549	3.70 × 10^3^
	PIGSENSEKTTMP (Mox)	1405.6395	1405.6472	1.90 × 10^7^
	IGSENSEKTTMP (Mox)	1308.5867	1308.5918	6.50 × 10^5^
β-casein	EPVLGPVRGPFPIIV	1588.9341	1588.9289	2.10 × 10^4^
	PVLGPVRGPFPIIV	1459.8915	1459.8866	5.20 × 10^6^
PIGR	AAPAGAAIQS	855.445	855.4435	1.90 × 10^3^
	AAPAGAAIQSR	1011.5461	1011.5526	4.40 × 10^4^

The calculated and measured masses are listed. The shown Expect values are from a representative experiment. Expect values below 0.05 are considered significant. Oxidation of methionine (Mox) is indicated. PIGR, polymeric immunoglobulin receptor. Only peptides identified in three out of five experiments (PBEC) are considered as transported and included in the table.

**Table 3:** Peptides identified on the basolateral side of Caco-2 and porcine brain endothelial cell (PBEC) monolayers after the synthetic peptide PIGSENSEKTTMPLW was added to the apical compartment.

Monolayer	Peptide (Basolateral)	Calculated	Measured	Expect
Caco-2	PIGSENSEKTTMP (Mox)	1405.6395	1405.6444	7.60 × 10^7^
	PIGSENSEKTT	1161.5513	1161.555	6.80 × 10^7^
PBEC	PIGSENSEKTTMP (Mox)	1405.6395	1405.6397	6.70 × 10^6^
	PIGSENSEKTT	1161.5513	1161.5586	2.30 × 10^6^

The calculated and measured masses are listed. The shown Expect values are from a representative experiment. Expect values < 0.05 are considered significant. Oxidation of methionine (Mox) is indicated.

## Supplementary Materials

The following are available online at https://www.mdpi.com/2072-6643/12/10/3157/s1, Table S1. All endogenous milk peptides identified by LC-MS/MS. Figure S1. MS/MS spectra of transported peptides from α_S1_-casein transport. Figure S2. Control in vitro gastrointestinal digestion of a synthetic osteopontin peptide and milk osteopontin.
