# Supplementary material for: Transport of a Peptide from Bovine αs1-Casein across Models of the Intestinal and Blood–Brain Barriers
Source: Nutrients. 2020 Oct 16;12(10):3157. doi: 10.3390/nu12103157 (PMC7602804; doi:10.3390/nu12103157)
Supplement: Supplementary file 1 [file nutrients-12-03157-s001.pdf]

Table S1: Peptides identified in bovine milk by LS-MS/MS.

Protein	Measured	Calculated	Sequence	Modification
Alpha-S1-casein	1070.5601	1070.5608	QEVLNENLL	
	870.4964	870.4923	LNENLLR	
	1089.585	1089.5859	VAPFPEVFGK	
	1218.6246	1218.6285	VAPFPEVFGKE	
	990.5185	990.5175	APFPEVFGK	
	1782.7496	1782.7465	DIGSESTEDQAMEDIK	OxMet
	885.4413	885.4444	EEIVPNSV	
	1051.5391	1051.5298	HIQKEDVPS	
	1199.6259	1199.6146	IQKEDVPSER	
	1007.5676	1007.5651	VPQLEIVPN	
	1579.8173	1579.8206	VPQLEIVPNSAEER	
	1659.7858	1659.7869	VPQLEIVPNSAEER	pSer
	1255.6416	1255.6408	LEIVPNSAEER	
	1335.6101	1335.6071	LEIVPNSAEER	pSer
	1142.5536	1142.5567	EIVPNSAEER	
	1222.5276	1222.5231	EIVPNSAEER	pSer
	1464.6112	1464.6068	VPNSAEERLHSM	OxMet/pSer
	1268.4911	1268.4856	NSAEERLHSM	OxMet/pSer
	1394.671	1394.6718	VPLGTQYTDAPSF	
	1658.7431	1658.7424	DAPSFSDIPNPIGSEN	
	1213.5985	1213.5979	APSFSDIPNPIG	
	1429.6689	1429.6725	APSFSDIPNPIGSE	
	1543.7151	1543.7154	APSFSDIPNPIGSEN	
	1759.7855	1759.7901	APSFSDIPNPIGSENSE	
	1485.6974	1485.6947	SDIPNPIGSENSEK	
	1844.8483	1844.8462	DIPNPIGSENSEKTTMP	OxMet
	939.4691	939.4661	IPNPIGSEN	
	1026.4998	1026.4982	IPNPIGSENS	
	1729.8229	1729.8192	IPNPIGSENSEKTTMP	OxMet
	1842.9046	1842.9033	IPNPIGSENSEKTTMPL	OxMet
	2028.9865	2028.9826	IPNPIGSENSEKTTMPLW	OxMet
	1161.5503	1161.5513	PIGSENSEKTT	
	1275.6037	1275.5943	NPIGSENSEKTT	
	1422.6335	1422.6297	NPIGSENSEKTTM	OxMet
	1519.682	1519.6824	NPIGSENSEKTTMP	OxMet
	1308.5929	1308.5867	PIGSENSEKTTM	OxMet
	1389.6447	1389.6446	PIGSENSEKTTMP	
	1405.645	1405.6395	PIGSENSEKTTMP	OxMet
	1518.7298	1518.7236	PIGSENSEKTTMPL	OxMet
	1688.8139	1688.808	PIGSENSEKTTMPLW	
	1704.8093	1704.8029	PIGSENSEKTTMPLW	OxMet
	1308.5935	1308.5867	IGSENSEKTTMP	OxMet
	1421.6494	1421.6497	SENSEKTTMPLW	

	1437.6466	1437.6446	SENSEKTTMPLW	OxMet
Alpha-S2-casein	2314.0111	2314.0271	KNTMEHVSSSEESIISQETY	OxMet
	1060.4901	1060.4842	IISQETYK	pSer
	1188.547	1188.5427	IISQETYKQ	pSer
	1212.4915	1212.4846	NMAINPSKEN	OxMet/pSer
	1038.5732	1038.571	NAVPITPTLN	
	1609.7016	1609.6947	KTVDMESTEVFTK	OxMet/pSer
	1257.5446	1257.5435	TVDMESTEVFT	
	1273.5385	1273.5384	TVDMESTEVFT	OxMet
	1401.6366	1401.6334	TVDMESTEVFTK	OxMet
	1465.6064	1465.6048	TVDMESTEVFTK	pSer
	1481.6093	1481.5997	TVDMESTEVFTK	OxMet/pSer
	1529.7305	1529.7283	TVDMESTEVFTKK	OxMet
	1609.6967	1609.6947	TVDMESTEVFTKK	OxMet/pSer
	1172.4928	1172.4907	VDMESTEVFT	OxMet
	1300.5869	1300.5857	VDMESTEVFTK	OxMet
	1380.5576	1380.552	VDMESTEVFTK	OxMet/pSer
	1073.4238	1073.4223	DMESTEVFT	OxMet
	1185.5276	1185.5224	DMESTEVFTK	
	1201.5201	1201.5173	DMESTEVFTK	OxMet
	1281.4861	1281.4836	DMESTEVFTK	pSer
	1086.4939	1086.4903	MESTEVFTK	OxMet
	1166.4599	1166.4566	MESTEVFTK	pSer
	939.458	939.4549	ESTEVFTK	
Beta-casein	1154.5959	1154.5931	RELEELNVPG	
	1127.5367	1127.5346	ELEELNVPGE	
	1555.762	1555.7617	ELEELNVPGEIVES	
	998.4945	998.492	LEELNVPGE	
	1313.6364	1313.6351	EELNVPGEIVES	
	1184.5922	1184.5925	ELNVPGEIVES	
	1107.5424	1107.5408	LSSSEESITR	
	1221.5902	1221.5837	SSSEESITRIN	
	1134.5492	1134.5517	SSEESITRIN	
	1047.5228	1047.5196	SEESITRIN	
	960.4896	960.4876	EESITRIN	
	831.4476	831.445	ESITRIN	
	1472.6552	1472.6548	KIEKFQSEEQQ	pSer
	1980.8644	1980.8548	FQSEEQQQTEDELQDK	
	2060.825	2060.8212	FQSEEQQQTEDELQDK	pSer
	1705.727	1705.7278	SEEQQQTEDELQDK	
	1618.6978	1618.6958	EEQQQTEDELQDK	
	1489.655	1489.6532	EQQQTEDELQDK	
	1089.5215	1089.519	TEDELQDKI	
	1299.6901	1299.6863	SLVYPFPGPIPN	
	991.5718	991.5702	LPQNIPPLT	
	1416.7975	1416.7977	LPQNIPPLTQTPV	

	967.5802	967.5743	TPVVVPPFL	
	1171.5955	1171.5947	VPPFLQPEVM	OxMet
	1327.6831	1327.6846	VPPFLQPEVMGV	OxMet
	1259.6852	1259.6907	GVSKVKEAMAPK	OxMet
	1219.5947	1219.5947	MPFPKYPVEP	OxMet
	1395.653	1395.6558	YPVEPFTESQSL	
	903.4573	903.4549	TLTDVENL	
	865.5057	865.5022	EPVLGPVR	
	1019.5775	1019.5764	EPVLGPVRGP	
	1263.6984	1263.6976	EPVLGPVRGPFP	
	1588.9331	1588.9341	EPVLGPVRGPFPIIV	
	1459.8939	1459.8915	PVLGPVRGPFPIIV	
	825.4532	825.4497	GPVRGPFP	
Kappa Casein	1917.9078	1917.8972	SPEVIESPPEINTVQVT	pSer
	2196.129	2196.1162	SPEVIESPPEINTVQVTSTAV	
	2276.0809	2276.0825	SPEVIESPPEINTVQVTSTAV	pSer
	1841.9251	1841.9258	EVIESPPEINTVQVTST	
	2012.0424	2012.0314	EVIESPPEINTVQVTSTAV	
	2092.0069	2091.9977	EVIESPPEINTVQVTSTAV	pSer
	1882.9932	1882.9888	VIESPPEINTVQVTSTAV	
	1183.6048	1183.6085	SPPEINTVQVT	
	1541.7979	1541.7937	SPPEINTVQVTSTAV	
	1454.7673	1454.7617	PPEINTVQVTSTAV	
	1131.6142	1131.6136	INTVQVTSTAV	
Butyrophilin	1699.6674	1699.6631	EGQEQEGEEMAEYR	OxMet
	1125.5334	1125.5302	VEDHIAEGSVA	
	1224.6014	1224.5986	VEDHIAEGSVAV	
	1026.4707	1026.4618	EDHIAEGSVA	
	897.4239	897.4192	DHIAEGSVA	
	1508.7173	1508.7107	IQEVKASDDGEYR	
	1212.5826	1212.5809	VAALGSDPHISM	OxMet
	1064.5151	1064.5138	DEEGLFTVR	
	973.446	973.4426	IPLSPMGED	OxMet
	1060.4767	1060.4747	IPLSPMGEDS	OxMet
	1846.8467	1846.8506	IPLSPMGEDSASGDIETL	OxMet
	1983.9154	1983.9095	IPLSPMGEDSASGDIETLH	OxMet
	1660.6962	1660.6886	SPMGEDSASGDIETLH	OxMet
	1476.6067	1476.6039	MGEDSASGDIETLH	OxMet
	1143.5085	1143.5044	DSASGDIETLH	
	941.4486	941.4454	ASGDIETLH	
	1034.5771	1034.576	IPLQPSQGVP	
Osteopontin	1437.5968	1437.5896	SNVQSPDATEEDF	
	1386.6236	1386.6263	ISHELDSASSEVN	
	1466.599	1466.5926	ISHELDSASSEVN	pSer
	1273.5427	1273.5422	SHELDSASSEVN	
	1186.514	1186.5102	HELDSASSEVN	

Beta-lactoglobulin	820.4387	820.4364	LIVTQTM	OxMet
	948.5325	948.5314	LIVTQTMK	OxMet
	1005.5529	1005.5529	LIVTQTMKG	OxMet
	1233.6669	1233.6639	LIVTQTMKGLD	OxMet
	915.468	915.4661	IDALNENK	
	1015.4374	1015.4346	PEVDDEALE	
	1193.5457	1193.5452	EVDDEALEKF	
	1064.5038	1064.5026	VDDEALEKF	
	965.437	965.4342	DDEALEKF	
	1196.5547	1196.5496	PTQLEEQCHI	
	1276.5124	1276.5159	PTQLEEQCHI	pSer
Mucin-1	1566.6854	1566.6798	SPYEEVSAGNGGSNLS	
	974.5037	974.5032	TNLAATSANL	
Polymeric immunoglobulin Receptor	1602.7806	1602.789	SPIFGPEEVTSVEGR	
	1011.544	1011.5461	AAPAGAAIQSR	
	940.5059	940.509	APAGAAIQSR	
	1438.7494	1438.7528	APAGAAIQSRAGEIQ	
	1170.6177	1170.6244	AGEIQNKALLD	
	1107.5923	1107.5964	ALLDPSFFAK	
	982.6063	982.6063	ALVSTLVPLA	
Glycosylation-dependent cell adhesion molecule 1	2178.0502	2178.044	ILNKPEDETHLEAQPTDASA	
	2306.0929	2306.1026	ILNKPEDETHLEAQPTDASAQ	
	2453.1795	2453.171	ILNKPEDETHLEAQPTDASAQF	
	2722.362	2722.3562	ILNKPEDETHLEAQPTDASAQFIR	
	1038.4641	1038.4618	DETHLEAQP	
	1127.533	1127.5319	SSRQPQSQNP	
	1291.7006	1291.6997	RQPQSQNPKLP	
Fatty acid synthase	1391.7826	1391.7885	RPLPVLGGNVGINS	
	1059.5171	1059.5171	APSPAAMPFR	OxMet
	1279.5954	1279.6019	APSPAAMPFRGY	OxMet
	982.4909	982.4971	SPIPETDPK	
	1069.5289	1069.5291	SPIPETDPKS	
	1182.6119	1182.6132	SPIPETDPKSL	
	1082.4867	1082.488	EEEQEAVLH	
Myosin regulatory light polypeptide 9	1057.5045	1057.504	TDPEDVIRN	
	956.4575	956.4563	DPEDVIRN	
Protein canopy homolog 3	948.5397	948.5392	LPAPELGPR	
	1147.6358	1147.6349	LPAPELGPRQA	
Zinc finger protein 526	994.5483	994.5488	PPVTPPPPPP	
	865.4625	865.4698	PPPAPPPPP	
60S ribosomal protein L27	974.4355	974.4305	IDDGTSDRP	
Beta-1.4-galactosyltransferase 1	955.5046	955.5087	IGQPSGELR	
V-type proton ATPase subunit a	864.4173	864.409	GQGHSPPSV	
Steroidogenic factor 1	853.4754	853.4698	GVPPPPPPP	
POLK protein	817.4179	817.4181	KEVNELS	

Guanine nucleotide-binding protein G(s) subunit alpha	1196.5648	1196.5673	TPEPGEDPRVT	
	998.4652	998.4669	EPGEDPRVT	
Serum amyloid A protein	1375.6048	1375.6078	AYQDMKEANYK	OxMet
Cystatin-C	952.51	952.509	VSPAAAQGPR	
Acyl-CoA desaturase	1033.4381	1033.4386	EEDIRPEM	OxMet
	1028.588	1028.5866	PAVKEKGSTL	
Constitutive coactivator of PPAR-gamma-like protein 1	838.4544	838.4549	IQPIPSQG	
Mucin-15	1491.6082	1491.6073	NPTANDSSTSAGGENA	
RAB1A, member RAS oncogene family	1489.7202	1489.7195	MGPGATAGGAEKSNVK	OxMet
ETS-related transcription factor Elf-5	1102.5153	1102.5142	NDPDETKATL	
Ribonuclease pancreatic	1229.6102	1229.6081	PYVPVHFDASV	
Hematological and neurological expressed 1 protein	887.4269	887.425	RPPGGGSNF	

Figure S1: MS/MS spectra of transported peptides.

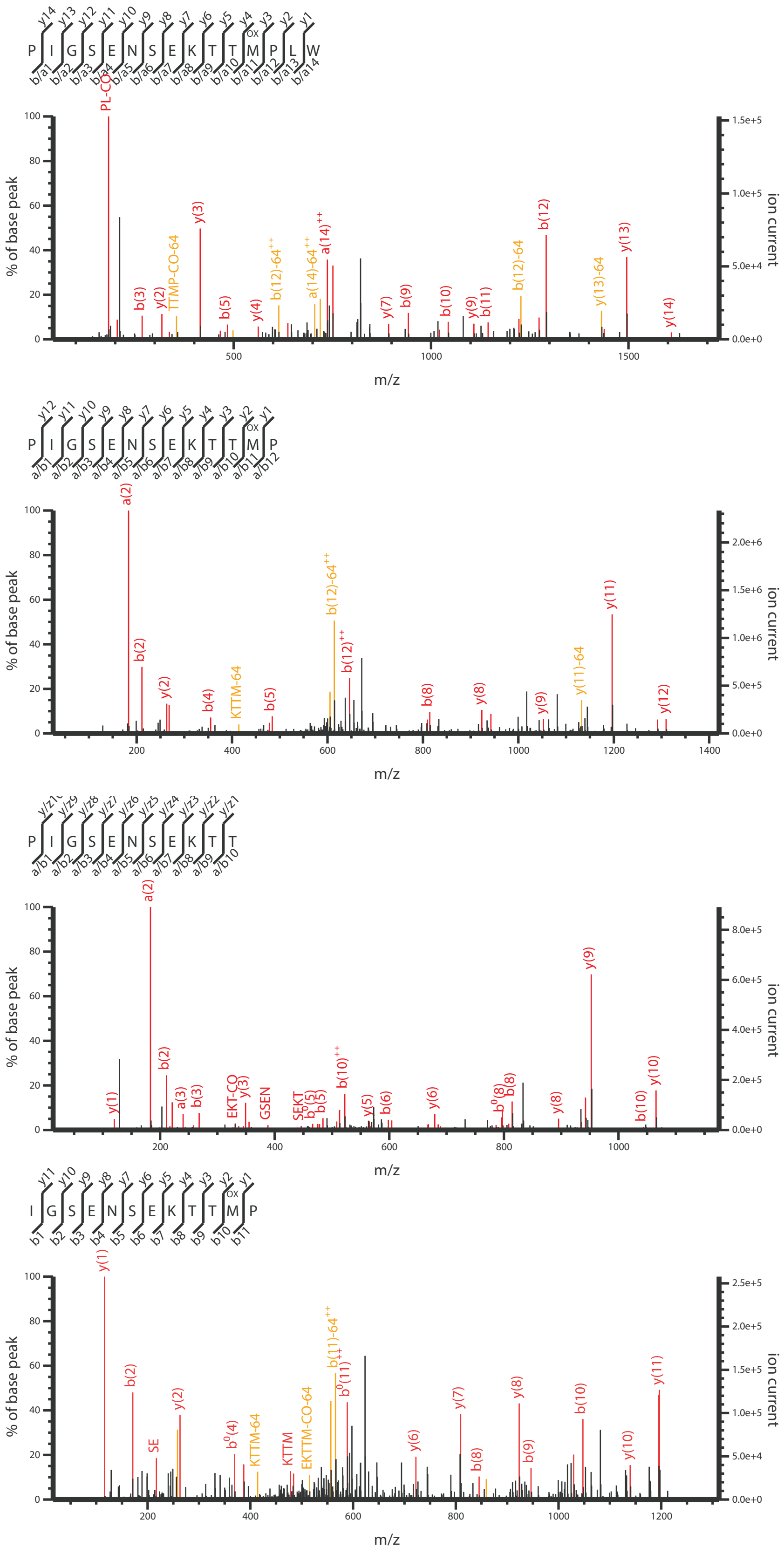

Figure S1: LC-MS/MS identified peptides from αS1-casein transported over Caco-2 monolayers. Representative spectra of peptides of PIGSENSEKTTM-PLW, PIGSENSEKTTMP, PIGSENSEKTT and IGSENSEKTTMPLW.

Figure S2: Control of the gastrointestinal digestion.

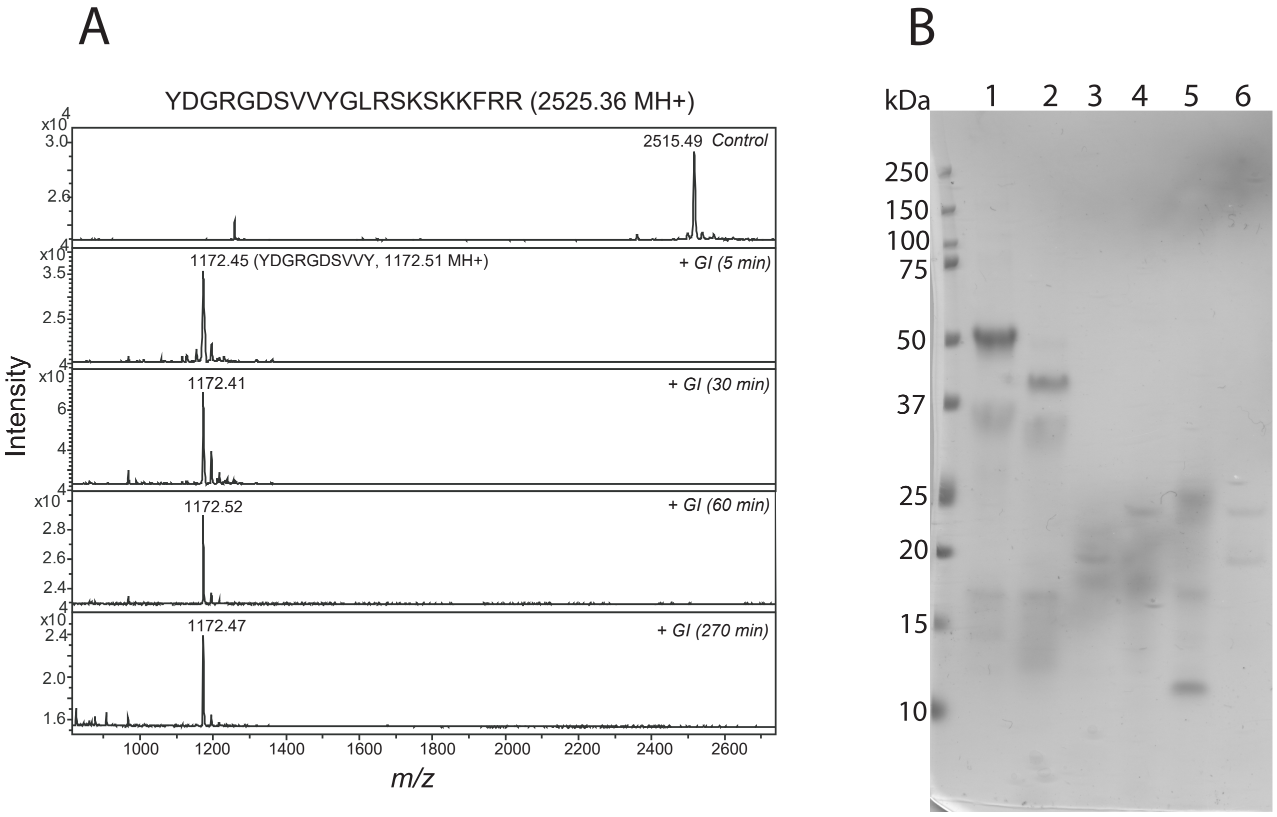

Figure S2. Control of the gastrointestinal digestion. A, simulated gastrointestinal digestion of the synthetic peptide, YDGRGDSVVYGLRSKSKKFRR, from the human osteopontin sequence. MS spectra of the untreated peptide, and the peptide subjected to gastrointestinal digestion for 5 min, 30 min, 60 min and 270 min, respectively. Resulting peptides were analysed by MALDI-MS and monoisotopic masses are assigned the individual peaks. Calculated value of the peptide is listed above the spectra. B, SDS-PAGE of untreated bovine milk osteopontin (lane 1), or osteopontin digested with pepsin (lane 2), trypsin (lane 3), chymotrypsin (lane 4), elastase (lane 5) or the protein digested with pepsin and subsequently trypsin, chymotrypsin and elastase (lane 6).
